# Effects of Sarcopenia on the Outcomes and Safety of Chemoradiotherapy Followed by Durvalumab for the Treatment of Patients With Locally Advanced Non‐Small Cell Lung Cancer

**DOI:** 10.1111/1759-7714.70145

**Published:** 2025-08-15

**Authors:** Kentaro Tamura, Hidehito Horinouchi, Mototaka Miyake, Ken Masuda, Yuki Shinno, Yusuke Okuma, Tatsuya Yoshida, Noboru Yamamoto, Yasushi Goto

**Affiliations:** ^1^ Department of Thoracic Oncology National Cancer Center Hospital Tokyo Japan; ^2^ Division of Respiratory Diseases, Department of Internal Medicine The Jikei University School of Medicine Tokyo Japan; ^3^ Department of Diagnostic Radiology National Cancer Center Hospital Tokyo Japan

**Keywords:** chemoradiation therapy, durvalumab, locally‐advanced non‐small cell lung cancer, sarcopenia

## Abstract

**Background:**

Sarcopenia is associated with poor outcomes of various cancers treated with immune checkpoint inhibitors. Durvalumab is the standard of care for patients with locally advanced (LA) non‐small cell lung cancer (NSCLC) after chemoradiation therapy (CRT). However, the effect of sarcopenia on the efficacy and safety of durvalumab in patients with LA‐NSCLC remains unclear.

**Methods:**

This single‐center retrospective study was conducted between 2018 and 2021. Body composition indices were measured using computed tomography scans taken at the third lumbar vertebra before and after CRT. The cutoff values were set based on the change ratios for each index before and after CRT. Tumor response, survival, and the efficacy and safety of durvalumab were compared between patients who showed skeletal muscle loss and those who did not.

**Results:**

Among 153 eligible patients (median age: 65 years; 74.5% men), skeletal muscle index (SMI) significantly decreased during CRT. With the threshold set at a −10% change in SMI, no significant difference in objective response rate (ΔSMI ≤ −10% vs. ΔSMI > −10%: 76.6% vs. 75.7%, *p* = 1.000), progression‐free survival (hazard ratio [HR], 0.99, *p* = 0.983), overall survival (HR 1.04, *p* = 0.909), or the frequency of immune‐related adverse events (44.9% vs. 44.2%, *p* = 1.000) was observed between the two groups.

**Conclusions:**

Although muscle loss during CRT is common, it does not compromise the efficacy or safety of subsequent durvalumab therapy in patients with LA‐NSCLC. Future studies are needed to delineate sarcopenia criteria specific to LA‐NSCLC and assess interventions, including rehabilitation and pharmacotherapy.

## Graphical abstract

Muscle loss during chemoradiotherapy is common in patients with locally advanced non‐small cell lung cancer. However, our retrospective study showed that this muscle loss does not negatively affect the efficacy or safety of subsequent durvalumab treatment.

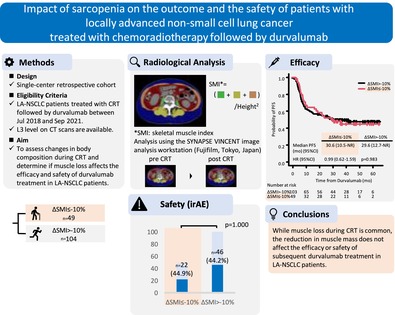

## 1 Introduction

Sarcopenia was first defined by Rosenberg as age‐related loss of lean tissue, especially skeletal muscle, which reduces the ability to perform activities of daily living and increases the risk of falls and fractures, particularly in the elderly [1]. However, sarcopenia can also occur as a secondary loss of muscle tissue associated with malignancies.

Unintentional weight loss of 5% or more predicts decreased survival and an increased risk of treatment‐related adverse events in several types of cancer [2]. In addition, malignancy‐associated sarcopenia has been identified as an independent prognostic factor in various cancers, including lung cancer [3, 4]. Furthermore, sarcopenic obesity is an independent prognostic factor for solid tumors [3]. Moreover, it has been reported that 46.8% of patients with stage III‐IV non‐small cell lung cancer (NSCLC) have sarcopenia [5, 6]. Therefore, weight loss and changes in body composition should be considered when predicting the clinical outcomes and safety of anti‐cancer therapies.

Immune checkpoint inhibitors (ICIs) have revolutionized the treatment paradigm for lung cancer, especially unresectable locally advanced (LA) NSCLC. Durvalumab, an anti‐programmed death‐ligand‐1 (PD‐L1) monoclonal antibody, has shown significant survival benefits compared with chemoradiotherapy (CRT) alone, establishing a new standard of care [7]. However, several meta‐analyses have indicated that ICIs exhibit reduced efficacy in patients with sarcopenia [8, 9]. Interleukin (IL)‐15 produced by skeletal muscle cells is associated with the proliferation of natural killer (NK) cells and T cells [10]. Sarcopenia leads to reduced NK and T cell activities, resulting in poor efficacy of ICI therapy [10]. Although it has been established that sarcopenia negatively affects the outcomes of ICIs in patients with metastatic cancers, its effect on the efficacy and safety of durvalumab in patients with LA‐NSCLC treated with CRT followed by durvalumab remains unclear [8, 9].

We conducted this study to analyze the changes in body composition during CRT and determine whether muscle loss during CRT affects the efficacy and safety of subsequent durvalumab therapy in patients with LA‐NSCLC. Understanding the effects of sarcopenia on durvalumab treatment could help identify patients with an increased risk of poor outcomes and inform decision‐making regarding the personalization of therapeutic strategies for improved prognosis and reduced risk of treatment‐related adverse events.

## 2 Methods

### 2.1 Study Design and Patients

This was a retrospective cohort study of consecutive patients with LA‐NSCLC who were treated with CRT followed by durvalumab between July 2018 and September 2021 at the National Cancer Center Hospital, Tokyo, Japan. Patients with oncogene‐addicted NSCLC or patients who showed local recurrence after surgery or stereotactic body radiation therapy and received curative CRT were eligible for inclusion in this study. Patients without computed tomography (CT) scans taken at the level of the third lumbar vertebra (L3) before and after CRT were excluded.

### 2.2 Data Collection

Data on baseline patient characteristics, including tumor histology, programmed cell death ligand‐1 (PD‐L1) status, driver oncogene status, clinical stage, Eastern Cooperative Oncology Group (ECOG) performance status (PS), and clinical outcomes before CRT and durvalumab administration, were extracted from the patients' electronic medical records (EMRs). Comorbidities were assessed using the Charlson Comorbidity Index [11], whereas cachexia was assessed using Evans' definition [12]. PD‐L1 expression levels were determined using PD‐L1 IHC 22C3 pharmDx (Agilent Technologies, Santa Clara, CA, USA). Driver oncogenes were identified using fluorescence in situ hybridization, immunohistochemistry, real‐time polymerase chain reaction‐based methods, or next‐generation sequencing. Data collection was conducted until the cut‐off date of February 29, 2024. Structured data, which included predefined, formatted, and coded data, were directly extracted from the patients' EMRs. KT collated unstructured data, such as information from written case notes or data that could not be extracted directly from patient records. All the extracted data were downloaded for analysis in this study. The data collection process depended on the availability, quality, and validity of the data. The extracted data were evaluated with due diligence to ensure conformity, completeness, and plausibility.

### 2.3 Radiological Analysis

The diagnostic criteria for sarcopenia were first proposed by the European Working Group on Sarcopenia in Older People in 2009. The criteria were focused on quantifying skeletal muscle and muscle function, including grip strength and walking speed [13]. The International Working Group on Sarcopenia and Asian Working Group for Sarcopenia have also established diagnostic criteria for sarcopenia, with the criteria by the latter group being particularly focused on Asians, including the Japanese [14, 15]. These criteria were established based on the skeletal muscle mass of healthy elderly people [16]. However, quantification of muscle mass requires specialized examination equipment, such as dual‐energy X‐ray absorptiometry (DXA) or bioelectrical impedance analysis (BIA). Notably, skeletal muscle mass observed on CT scans taken at the L3 level is correlated with the skeletal muscle mass calculated using DXA and BIA [17, 18]. In several recent studies, assessment of sarcopenia was performed by measuring skeletal muscle mass at the L3 level using CT scans [3, 4].

**FIGURE 1:**
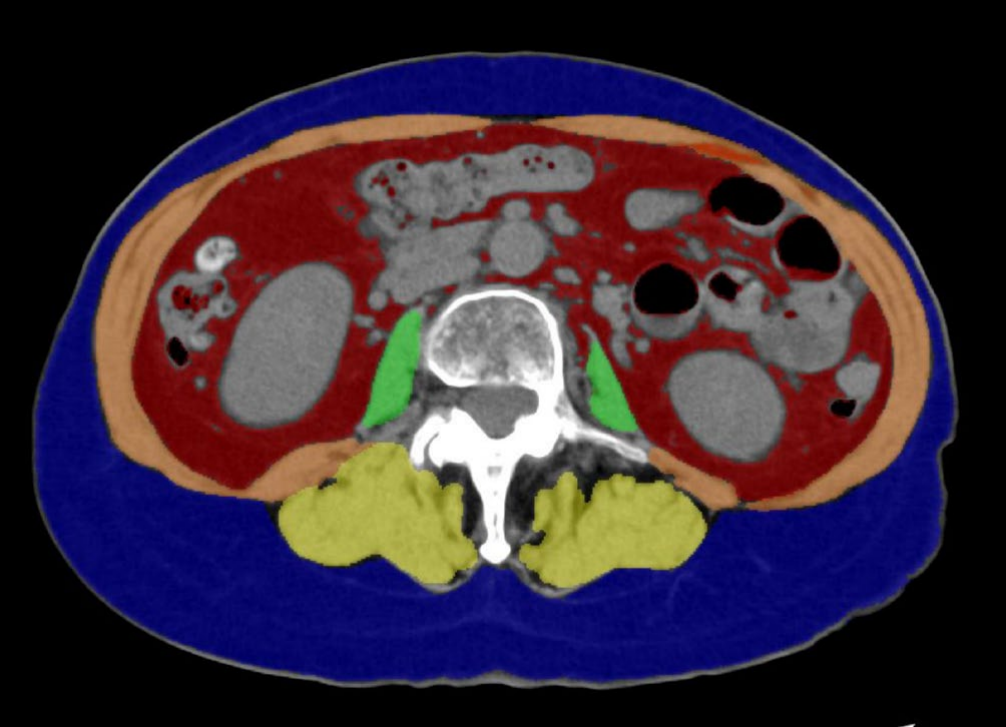
Body composition indices in computed tomography images taken at the level of the third lumbar vertebra. The green, orange, and yellow areas represent skeletal muscles. Specifically, the green area indicates the psoas muscle, the red area represents visceral adipose tissue, and the blue area represents subcutaneous adipose tissue.

KT conducted image analyses using the SYNAPSE VINCENT image analysis workstation (Fujifilm, Tokyo, Japan) at our hospital. The data used for the image analyses were obtained from CT scans taken within 28 days before the start of CRT, 10 days after CRT, and 28 days before the start of durvalumab. Contours were automatically extracted by setting the Hounsfield unit (HU) threshold within a specific range. The HU thresholds were set as follows based on the information in previous studies: skeletal muscle and iliopsoas muscle, −29 to +150; visceral adipose tissue, −150 to −50; and subcutaneous adipose tissue, −190 to −30 [3, 4]. Regions within the automatically extracted contours that were clearly not targeting muscle or adipose tissue were manually deleted. Examples of the muscle and adipose tissues are shown in Figure 1. These four areas (cm^2^) were divided by the height (m) squared to obtain the skeletal muscle index (SMI), psoas muscle index (PMI), visceral adipose index (VAI), and subcutaneous adipose index (SAI). There is currently no consensus on the cutoff values for muscle mass indices related to sarcopenia, especially in Japanese patients with LA‐NSCLC. Therefore, the cutoff values were set based on the change ratios for each index before and after CRT.

### 2.4 Efficacy and Safety

Tumor response was assessed according to the Response Evaluation Criteria in Solid Tumors version 1.1 [19]. Immune‐related adverse events (irAEs) were graded according to the National Cancer Institute Common Terminology Criteria for Adverse Events, version 5.0 [20]. Data on irAEs observed from the start date of durvalumab treatment to the cut‐off date were extracted from the patients' EMRs.

### 2.5 Variables

The overall response rate (ORR) was defined as the proportion of patients who achieved the best overall response, either complete or partial. Progression‐free survival (PFS) and overall survival (OS) curves were generated using the Kaplan–Meier method. PFS was defined as the time from the start of durvalumab treatment to disease progression or death from any cause. Patients without disease progression were censored at the last follow‐up visit. OS was defined as the time from the start of durvalumab therapy to death from any cause. Patients who died were censored at the last follow‐up visit.

### 2.6 Statistical Analysis

Changes in albumin level, hemoglobin level, C‐reactive protein (CRP) level, body weight, body mass index (BMI), SMI, PMI, VAI, and SAI before and after CRT were compared using the Wilcoxon signed‐rank test. The correlations between SMI, BMI, and PMI were analyzed using Spearman's rank correlation coefficient. Cutoff values were set based on decrease in SMI during CRT. Prognostic factors were compared between patients who showed decreased SMI and those who did not. PFS and OS curves were plotted using the Kaplan–Meier method. Hazard ratios (HRs) and 95% confidence intervals (CIs) were calculated using the Cox proportional hazards model. To control for confounding factors, Cox proportional hazards model analysis was performed using variables identified as significant in the univariate analysis. Missing values were omitted from the analysis. Statistical significance was set at *p* < 0.05 (two‐sided). All statistical procedures were performed using the EZR (Easy R) statistical software version 1.54 [21].

### 2.7 Ethical Considerations

This study was approved by the Ethics Committee of the National Cancer Center Hospital (2021‐379). Details of the study were made available to the public, and patients were provided the opportunity to opt out of participation.

## 3 Results

### 3.1 Patient Characteristics

**FIGURE 2:**
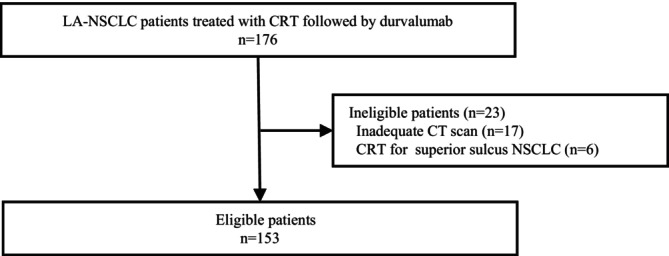
Patient selection flowchart. CRT, chemoradiation therapy; LA, locally advanced; NSCLC, non‐small cell lung cancer.

Of 176 patients with LA‐NSCLC who received CRT followed by durvalumab during the study period, 153 were included in this study (Figure 2). The characteristics of the patients at the start of durvalumab treatment are summarized in Table 1. The median age of the patients was 65 years (interquartile range [IQR], 56.0–72.0); 114 (74.5%) of them were men, 131 (85.6%) had a smoking history, 16 (10.5%) had a CCI of 3 points or higher, 63 (41.4%) met criteria for cachexia, 91 (59.5%) had adenocarcinoma, 17 (11.1%) had *epidermal growth factor receptor* (*EGFR*) mutations, 6 (3.9%) tested positive for *anaplastic lymphoma kinase* (*ALK*) fusion gene, 49 (32.0%) had a PD‐L1 expression level of 50% or higher, 6 (3.9%) had stage II disease, 126 (82.4%) had stage III disease, and 21 (13.7%) showed recurrence.

**TABLE 1:** Baseline patient characteristics before durvalumab treatment.

*n* (%)	Total (*n* = 153)
Age (years), median (IQR)	65.0 (56.0, 72.0)
≥ 75	28 (18.3)
Sex	
Men	114 (74.5)
Women	39 (25.5)
Smoking history	
Never	22 (14.4)
Ex or current	131 (85.6)
Charlson comorbidity index	
0–2	137 (89.5)
≥ 3	16 (10.5)
Cachexia	63 (41.4)
Histology	
Adenocarcinoma	91 (59.5)
Squamous	42 (27.4)
NSCLC, NOS	20 (13.1)
Oncogenic driver genes	
*EGFR*	
Negative	96 (62.8)
19del/L858R	13 (8.5)
Uncommon	4 (2.6)
NE	40 (26.1)
*ALK*	
Negative	103 (67.3)
Positive	6 (3.9)
NE	44 (28.8)
PD‐L1 TPS (%)	
< 1	37 (24.2)
1–49	39 (25.5)
≥ 50	49 (32.0)
NE	28 (18.3)
Stage	
IIA/IIB	2 (1.3)/4 (2.6)
IIIA/IIIB/IIIC	54 (35.3)/55 (36.0)/17 (11.1)
Recurrence	21 (13.7)
ECOG‐PS	
0	62 (40.5)
1	91 (59.5)
Chemotherapy regimen	
CDDP doublet	119 (77.8)
CBDCA doublet	28 (18.3)
CBDCA	6 (3.9)
Radiation dose (Gy), median (IQR)	60 (60, 60)
Interval between CRT and durvalumab (day), median (IQR)	18 (14, 24)

Abbreviations: ALK, anaplastic lymphoma kinase; CBDCA, carboplatin; CDDP, cisplatin; CRT, chemoradiotherapy; del, exon 19 deletion; ECOG‐PS, Eastern Cooperative Oncology Group performance status; EGFR, epidermal growth factor receptor; Gy, gray; IQR, interquartile range; L858R, exon 21 on L858R mutation; NE, not evaluated; NOS, not otherwise specified; NSCLC, non‐small cell lung cancer; PD‐L1 TPS, programmed cell death‐ligand tumor proportion score.

### 3.2 Changes in Body Composition and Laboratory Values During Chemoradiotherapy

The men exhibited decreased body weight and significantly decreased BMI during CRT ([pre CRT vs. post CRT] body weight [median, IQR] [kg]: 65.2 [57.1, 72.1] vs. 62.0 [54.4, 68.9], *p* = 0.059; BMI [kg^2^/m^2^]: 23.2 [20.5, 25.2] vs. 22.3 [19.7, 24.4], *p* = 0.047) (Table 2). However, the women showed a non‐significant decrease in both body weight and BMI (body weight: 50.7 [46.0, 59.6] vs. 49.6 [45.0, 56.3], *p* = 0.308; BMI: 20.7 [18.9, 23.0] vs. 19.8 [18.4, 21.3], *p* = 0.287) (Table 2). Men also showed a significant decrease in SMI and PMI during CRT (SMI [cm^2^/m^2^]: 46.5 [41.6, 50.7] vs. 43.0 [38.9, 47.4], *p* = 0.001; PMI [cm^2^/m^2^]: 5.34 [4.40, 6.09] vs. 4.97 [4.20, 5.76], *p* = 0.044) (Table 2). The women showed a significant decrease in SMI during CRT (38.1 [33.9, 42.5] vs. 35.1 [32.3, 37.6], *p* = 0.029), and a non‐significant decrease in PMI (3.51 [3.01, 4.14] vs. 3.21 [2.63, 3.93], *p* = 0.257) (Table 2). Furthermore, the patients showed a non‐significant increase in VAI during CRT (Table 2). No change in SAI was observed during CRT (Table 2).

**TABLE 2:** Changes in body composition and laboratory results during chemoradiotherapy.

Body composition values	Sex	Pre‐CRT median (IQR)	Post‐CRT median (IQR)	Absolute difference	Relative difference (%)	*p*
Body weight (kg)	Men	65.2 (57.1, 72.1)	62.0 (54.4, 68.9)	−3.2	−4.9	0.059
	Women	50.7 (46.0, 59.6)	49.6 (45.0, 56.3)	−1.1	−2.2	0.308
BMI (kg^2^/m^2^)	Men	23.2 (20.5, 25.2)	22.3 (19.7, 24.4)	−0.9	−3.9	0.047
	Women	20.7 (18.9, 23.0)	19.8 (18.4, 21.3)	−0.9	−4.3	0.287
SMI (cm^2^/m^2^)	Men	46.5 (41.6, 50.7)	43.0 (38.9, 47.4)	−3.5	−7.5	0.001
	Women	38.1 (33.9, 42.5)	35.1 (32.3, 37.6)	−3.0	−7.9	0.029
PMI (cm^2^/m^2^)	Men	5.34 (4.40, 6.09)	4.97 (4.20, 5.76)	−0.37	−6.9	0.044
	Women	3.51 (3.01, 4.14)	3.21 (2.63, 3.93)	−0.30	−8.5	0.257
VAI (cm^2^/m^2^)	Men	39.0 (16.5, 56.6)	37.9 (17.1, 51.0)	−1.1	−2.8	0.432
	Women	19.5 (10.0, 33.0)	16.6 (9.7, 30.6)	−2.9	−14.9	0.664
SAI (cm^2^/m^2^)	Men	31.8 (17.7, 44.1)	31.7 (17.7, 43.7)	−0.1	−0.3	0.805
	Women	32.7 (22.6, 46.5)	32.7 (20.6, 54.1)	0.0	0.0	0.830

Laboratory data	Pre‐CRT median (IQR)	Post‐CRT median (IQR)	Absolute difference	Relative difference (%)	*p*
Alb (g/dL)	4.1 (3.7, 4.3)	3.9 (3.6, 4.1)	−0.2	−4.9	0.001
Hb (g/dL)	13.3 (12.4, 14.3)	11.1 (10.2, 12.3)	−2.2	−16.5	< 0.001
CRP (mg/dL)	0.19 (0.06, 0.90)	0.18 (0.07, 0.66)	−0.01	−5.3	0.911

Abbreviations: Alb, albumin; BMI, body mass index; CRP, C‐reactive protein; CRT, chemoradiation therapy; Hb, hemoglobin; IQR, interquartile range; PMI, psoas muscle index; SAI, subcutaneous adipose index; SMI, skeletal muscle index; VAI, visceral adipose index.

Pre‐CRT SMI was strongly correlated with body weight (*ρ* = 0.75, *p* < 0.001), BMI (*ρ* = 0.73, *p* < 0.001), and PMI (*ρ* = 0.73, *p* < 0.001) (Figure S1A–C). Similarly, the SMI recorded before durvalumab treatment was strongly correlated with body weight (*ρ* = 0.73, *p* < 0.001), BMI (*ρ* = 0.69, *p* < 0.001), and PMI (*ρ* = 0.74, *p* < 0.001) (Figure S1D–F). A cutoff value of −10% change in SMI and PMI during CRT was set for both men and women. A 10% or higher decrease in SMI or PMI was observed in 49 (32.0%) and 42 (27.5%) patients, respectively (Table S1). A 5% or higher decrease in body weight loss was observed in 56 (36.6%) patients (Table S1). Although there were no differences in these factors between sexes, a BMI of less than 20 was significantly more common in women than in men (men: 30 [26.5%] vs. women: 21 [53.8%], *p* = 0.003) (Table S1).

Regarding laboratory findings, the patients showed significantly decreased albumin (Alb) and hemoglobin (Hb) levels during CRT (Alb [g/dL]: 4.1 [3.7, 4.3] vs. 3.9 [3.6, 4.1], *p* = 0.001; Hb [g/dl]: 13.3 [12.4, 14.3] vs. 11.1 [10.2, 12.3], *p* < 0.001). However, no change in C‐reactive protein (CRP) levels was observed during CRT (Table 2).

### 3.3 Therapeutic Efficacy in the Entire Cohort

The ORR for durvalumab was 76.0% (95% CI, 66.4–84.0) (Table S2). The median follow‐up period in this study was 40.7 months (95% CI, 36.1–45.0). The median PFS and OS were 29.6 months (95% CI, 17.5–not reached) and not reached (95% CI, not reached–not reached), respectively (Figure S2A,B). In subgroup analyses of PFS and OS, no significant differences were observed across all subgroups except in patients with squamous cell carcinoma (PFS: HR, 1.23 [95% CI, 0.76–1.99], *p* = 0.400; OS: HR, 1.96 [95% CI, 1.11–3.47], *p* = 0.022). However, the following factors were associated with a worse prognosis: age 75 years or older (PFS: HR, 1.28 [95% CI, 0.74–2.20], *p* = 0.363; OS: HR, 1.75 [95% CI, 0.91–3.35], *p* = 0.092), BMI of 20 or higher (PFS: HR, 1.63 [95% CI, 0.98–2.72], *p* = 0.059; OS: HR, 1.31 [95% CI, 0.71–2.42], *p* = 0.398), *EGFR* mutation or a positive *ALK* status (PFS: HR, 1.61 [95% CI, 0.93–2.79], *p* = 0.090; OS: HR, 0.67 [95% CI, 0.29–1.56], *p* = 0.351), a negative PD‐L1 status (PFS: HR, 1.50 [95% CI, 0.91–2.46], *p* = 0.105; OS: HR, 1.36 [95% CI, 0.73–2.53], *p* = 0.331), and stage III disease (PFS: HR, 1.55 [95% CI, 0.82–2.93], *p* = 0.180; OS: HR, 1.44 [95% CI, 0.65–3.19], *p* = 0.376) (Figure S2C,D). In contrast, factors related to sarcopenia, including a BMI of less than 20, weight loss of 5% or higher, cachexia, or decrease in SMI or PMI of 10% or higher, had no effects on prognosis (Figure S2C,D).

### 3.4 Characteristics of Patients Who Showed Decreased SMI During CRT and the Effects of Decreased SMI on the Efficacy of Durvalumab

The cohort of patients who showed decreased SMI (ΔSMI ≤ −10%) were generally older (ΔSMI ≤ −10% vs. ΔSMI > −10%: 67.0 [56.0, 72.0] vs. 64.0 [56.0, 72.0], *p* = 0.315), had more patients with stage II or recurrent disease (38 [77.6%] vs. 88 [84.6%], *p* = 0.363), and more patients with poorer PS (≥ 2) (1 [2.0%] vs. 0 [0.0%], *p* = 0.320) than the patients who did not show decreased SMI (ΔSMI > −10%) (Table S3). However, there was no significant difference in patient characteristics, including comorbidities and the presence of cachexia, between the two groups (Table S3).

**FIGURE 3:**
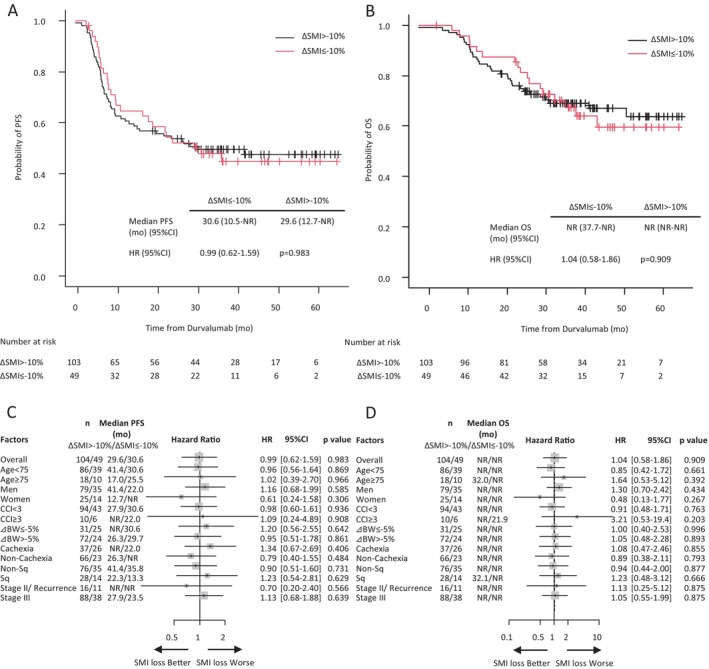
Survival outcomes of patients who showed skeletal muscle loss during chemoradiation therapy and patients who did not. Kaplan–Meier estimates of progression‐free survival (PFS) (A) and overall survival (OS) (B) are shown for patients who showed skeletal muscle loss of 10% or higher (ΔSMI ≤ −10%) during CRT and those who did not show significant skeletal muscle loss (ΔSMI > −10%). PFS (C) and OS (D) subgroup analyses. CRT, chemoradiation therapy; BW, body weight loss of 5% or higher (ΔBW ≤ −5%) during CRT; CCI, Charlson Comorbidity Index; CI, confidence intervals; HR, hazard ratio; NR, not reached; SMI, skeletal muscle index, calculated as skeletal muscle area divided by height squared; Sq, squamous cell carcinoma. Cachexia was assessed using Evans' definition.

**FIGURE 4:**
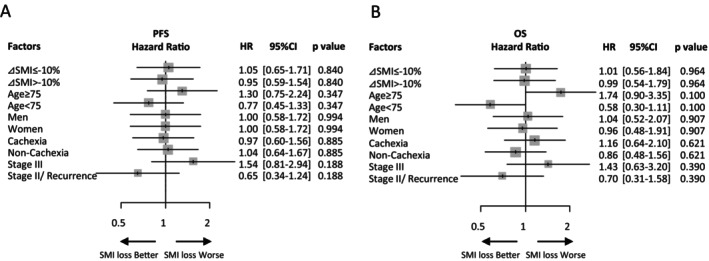
Multivariate analysis of survival outcomes of patients who showed skeletal muscle loss during chemoradiation therapy and patients who did not. Forest plot illustrating progression‐free survival (PFS) (A) and overall survival (OS) (B). CI, confidence interval; HR, hazard ratio; SMI, skeletal muscle index, calculated as skeletal muscle area divided by height squared. Patients are categorized based on whether they showed skeletal muscle loss of 10% or higher (ΔSMI ≤ −10%) or no skeletal muscle loss (ΔSMI > −10%) during CRT.

There was no difference in the ORR for durvalumab between the patients who showed decreased SMI and those who did not (76.6% [95% CI, 62.0–87.7] vs. 75.7% [95% CI, 66.3–83.6], *p* = 1.000) (Table S2). However, the median PFS and OS of the two groups were similar (PFS: 30.6 vs. 29.6 months, HR 0.99 [95% CI, 0.62–1.59], *p* = 0.983; OS: not reached vs. not reached, HR 1.04 [95% CI, 0.58–1.86], *p* = 0.909) (Figure 3A,B). Univariate analyses of the PFS and OS of patients who showed decreased SMI and those who did not (Figure 3C,D) revealed non‐significant differences in PFS and OS between the two groups. However, women who showed decreased SMI tended to have better PFS and OS than those who did not (PFS: HR, 0.61 [95% CI, 0.24–1.58], *p* = 0.306; OS: HR, 0.48 [95% CI, 0.13–1.77], *p* = 0.267). In contrast, patients aged 75 or older and those with a CCI of 3 or higher tended to have worse OS (HR, 3.21 [95% CI, 0.53–19.4], *p* = 0.203), whereas patients with cachexia (HR, 1.34 [95% CI, 0.67–2.69], *p* = 0.406), and patients with stage III tended to have worse PFS (HR, 1.13 [95% CI, 0.68–1.88], *p* = 0.639). Multivariate analyses of the correlations between these abovementioned factors and PFS and OS were performed (Figure 4A,B). The results revealed a trend toward worse PFS and OS in patients aged 75 years or older (PFS: HR, 1.30 [95% CI, 0.75–2.24], *p* = 0.347; OS: HR, 1.74 [95% CI, 0.90–3.35], *p* = 0.100) and in patients with stage III disease (PFS: HR, 1.54 [95% CI, 0.81–2.94], *p* = 0.188; OS: HR, 1.43 [95% CI, 0.63–3.20], *p* = 0.390). However, decreased SMI was not a prognostic factor for PFS and OS.

### 3.5 Effect of Decreased SMI During CRT on the Safety of Durvalumab

The frequency of irAEs related to durvalumab therapy was not different between patients who showed decreased SMI and those who did not (ΔSMI ≤ −10% vs. ΔSMI > −10%: 22 (44.9%) vs. 46 (44.2%), *p* = 1.000) (Table S4). Additionally, the frequency of irAEs that required steroids was not significantly different between the two groups (ΔSMI≤ −10% vs. ΔSMI> −10%: 12 (24.5%) vs. 35 (33.7%), *p* = 0.268) (Table S4).

## 4 Discussion

In the present study, approximately 30% of the patients with LA‐NSCLC exhibited skeletal muscle loss during CRT. However, our results suggested that the muscle loss did not compromise the efficacy or safety of the subsequent durvalumab therapy.

Sarcopenia has been consistently shown to negatively affect the efficacy of ICIs [9, 22, 23, 24, 25, 26, 27, 28, 29, 30, 31, 32]. Unexpectedly, our results did not indicate an association between sarcopenia and a poor prognosis. One possible explanation for this finding is that our study population consisted of patients with a generally favorable clinical background. The PFS observed in the present study was more favorable than that reported in the PACIFIC trial, suggesting that our patients had more favorable prognostic factors [7]. Our study also included more patients with early‐stage disease or post‐surgical recurrent disease and fewer patients with squamous cell carcinoma, all of which are features associated with better outcomes [7]. Our study population is younger, has a higher proportion of female patients, more patients who showed postoperative or post‐radiation recurrence, and fewer patients with squamous cell carcinoma than previous studies on the prognostic impact of sarcopenia in patients with LA‐NSCLC [30, 31, 32]. These favorable characteristics may have contributed to the lack of an observed prognostic effect of sarcopenia in the present study. Notably, although CCI data were not included in previous studies, we incorporated comorbidity status into our analysis. Most of our patients had low CCIs, indicating a lower comorbidity burden. Although patients with both a CCI of 3 or higher and decreased SMI showed a trend toward worse OS, the relatively low comorbidity burden may have lessened the negative prognostic effect of sarcopenia. On the other hand, patients aged 75 years or older or those with stage III disease tended to experience poorer outcomes associated with loss of SMI during CRT. These vulnerable populations may be more susceptible to the adverse effects of sarcopenia, warranting further investigation. Overall, these findings suggest that sarcopenia may have limited prognostic relevance in patients with favorable baseline characteristics.

Chronic inflammation and inadequate energy supply, which are associated with sarcopenia, are believed to impair the efficacy of ICIs [33]. However, the patients included in the present study showed no signs of elevated inflammation, such as increased CRP levels, during CRT. Furthermore, the patients did not have advanced cancer, suggesting an absence of chronic inflammation. Cancer‐associated sarcopenia is typically driven by chronic inflammation. In contrast, treatment‐related sarcopenia may have a distinct etiology, including direct muscle damage caused by therapeutic agents, muscle wasting due to nausea, and anorexia caused by adverse events [34]. The platinum‐containing therapeutic agents administered to the patients in the present study are particularly known for causing treatment‐related sarcopenia [35]. However, advances in palliative care and radiation therapy techniques may have contributed to reducing the severity of adverse events.

Previous studies have indicated that patients with advanced cancer and sarcopenia are more likely to experience adverse events caused by cytotoxic anticancer agents [35]. This etiology is likely attributable to the common practice of determining chemotherapy dosages based on body surface area (BSA), which is calculated using height and weight without considering the influence of fat mass [35]. Therefore, obese patients may receive an overdose if the chemotherapy dosage is based on BSA [35]. In contrast, ICIs, which are monoclonal antibodies, exhibit linear pharmacokinetics that are independent of BSA, leading to fixed dosing. From a pharmacokinetic perspective, sarcopenia is unlikely to affect irAEs. Instead, sarcopenia likely induces an immunosuppressive state, thereby potentially reducing the frequency of irAEs. Previous studies have shown that reduction in muscle mass does not affect the frequency of irAEs, which is consistent with the findings of the present study [9, 36]. In contrast, a previous study has indicated that obesity in women is associated with an increased frequency of irAEs [9]. However, no effect of subcutaneous fat, visceral fat, or sex on the frequency of irAEs was observed in the present study (Table S5).

This study has several limitations. First, our cohort comprised a higher proportion of patients with favorable prognostic characteristics compared with previous studies [7, 30, 31, 32]. As mentioned above, this may have mitigated the negative impact of sarcopenia, and caution is warranted when extrapolating our findings to real‐world clinical practice. Second, it was a single‐center, retrospective study with a small sample size. Therefore, significant selection bias may have been introduced into the study. Third, the Japanese criteria for sarcopenia were established based on healthy elderly individuals; criteria for patients with LA‐NSCLC have not been validated [37]. Therefore, we focused on changes in muscle mass during CRT in our analysis. A similar study indicated that 36.7% of patients with cancer experienced a reduction in PMI of 10% or higher during CRT, which aligns with our findings and supports the validity of this study [31]. However, further studies are required to validate the criteria for sarcopenia in patients with LA‐NSCLC. Fourth, we did not assess the effect of muscle mass loss during CRT on quality of life or functional status. Anamorelin, a ghrelin receptor agonist, has been shown to improve lean body mass and was the first drug approved in Japan for the treatment of sarcopenia [38, 39]. Future research is needed to determine whether interventions such as anamorelin or rehabilitation may help mitigate the adverse effects of muscle mass loss during CRT.

In conclusion, this study demonstrated that although muscle mass loss during CRT is common in patients with LA‐NSCLC, the reduction in muscle mass does not affect the efficacy or safety of subsequent durvalumab treatment.

## Author Contributions

**K.T.:** conceptualization, methodology, investigation, formal analysis, writing – original draft preparation. **H.H., M.M., K.M., Y.S., Y.O., T.Y., N.Y.:** conceptualization, methodology, writing, review, and editing. **Y.G.:** conceptualization, methodology, writing – review and editing, and supervision.

## Conflicts of Interest

The authors declare the following financial interests and/or personal relationships that may be considered potential competing interests: Dr. Tamura and Dr. Miyake have nothing to disclose. Dr. Horinouchi reports grants from MSD, AbbVie, AstraZeneca, BMS, Ono, Merck Biopharma, Daiichi Sankyo, Janssen, Genomic Health, Chugai, Roche, and Novartis; and personal fees from MSD, AstraZeneca, BMS, Ono, Chugai, Roche, Novartis, Eli Lilly, and Kyowa Kirin, outside the submitted work. Dr. Masuda reports personal fees from Ono, AstraZeneca, Chugai, and Bristol Myers Squibb, outside the submitted work. Dr. Shinno reports grants from Ono, Janssen, and Japan Clinical Research Operation K.K.; and personal fees from BMS, Chugai, AstraZeneca, Eli Lilly, and Ono, outside the submitted work. Dr. Okuma reports grants from Roche and AbbVie; and personal fees from AstraZeneca, Eli Lilly, BMS, Pfizer, Nippon Boehringer Ingelheim, Chugai, Ono, and Taiho, outside the submitted work. Dr. Yoshida reports grants from Amgen, AstraZeneca, Takeda, Daiichi Sankyo, Ono, MSD, AbbVie, Novartis, Chugai, and BMS; and personal fees from Amgen, AstraZeneca, Ono, MSD, Novartis, Chugai, BMS, Taiho, Lilly, Roche, and ArcherDX, outside the submitted work. Dr. Yamamoto reports grants from Chugai, Taiho, Eisai, Quintiles, Astellas, BMS, Novartis, Daiichi Sankyo, Pfizer, Boehringer Ingelheim, Kyowa Hakko Kirin, Bayer, Ono, Takeda, Janssen, MSD, Merck, GSK, Sumitomo Dainippon, Chiome Bioscience Inc., Otsuka, Carna Biosciences, Genmab, and Shionogi; and personal fees from Chugai, Eisai, Lilly, BMS, Pfizer, Boehringer Ingelheim, Ono, Takeda, Sysmex, Otsuka, AstraZeneca, and CIMIC, outside the submitted work. Dr. Goto reports grants from AZK, Pfizer, AbbVie, Eli Lilly, BMS, Ono, Novartis, Kyorin, Daiichi Sankyo, and Preferred Networks; and personal fees from Pfizer, Eli Lilly, BMS, Ono, Kyorin, Daiichi Sankyo, Chugai, Taiho, Boehringer Ingelheim, MSD, Merck, Thermo Fisher, AstraZeneca, Chugai, Guardant Health Inc., and Illumina, outside the submitted work.

## Supporting information


**Figure S1:** Correlations between skeletal muscle index and body weight, body mass index, and psoas muscle index before chemoradiation therapy or durvalumab treatment. BMI, body mass index; PMI, psoas muscle index; SMI, skeletal muscle index.

**Figure S2:** Survival outcomes for patients with locally‐advanced non‐small cell lung cancer treated with chemoradiation therapy followed by durvalumab. Kaplan–Meier estimates of progression‐free survival (PFS) (A) and overall survival (OS) (B) in the entire cohort. PFS (C) and OS (D) subgroup analyses. ALK, anaplastic lymphoma kinase; BMI, body mass index; BW, body weight loss of 5% or higher (ΔBW ≤ −5%) during CRT; CCI, Charlson Comorbidity Index; CI, confidential intervals; EGFR, epidermal growth factor receptor; HR, hazard ratio; NR, not reached; PD‐L1, programmed death‐ligand 1; PMI, psoas muscle index, calculated as psoas muscle area divided by height squared; SMI, skeletal muscle index, calculated as skeletal muscle area divided by height squared; Sq, squamous cell carcinoma. Cachexia was assessed using Evans' definition.

**Table S1:** Changes in body composition during chemoradiotherapy.
**Table S2:** Best responses to durvalumab treatment.
**Table S3:** Comparison of pre‐chemoradiotherapy characteristics between patients who showed decreased SMI during chemoradiotherapy and patients who did not.
**Table S4:** Summary of immune‐related adverse events in patients who showed skeletal muscle loss during chemoradiation therapy and patients who did not.
**Table S5:** Summary of immune‐related adverse events categorized according to subcutaneous adipose tissue index, visceral adipose tissue index, and sex.

## Data Availability

The data that support the findings of this study are available from the corresponding author upon reasonable request.
