# Supplementary material for: Effects of Sarcopenia on the Outcomes and Safety of Chemoradiotherapy Followed by Durvalumab for the Treatment of Patients With Locally Advanced Non‐Small Cell Lung Cancer
Source: Thorac Cancer. 2025 Aug 15;16(16):e70145. doi: 10.1111/1759-7714.70145 (PMC12355038; doi:10.1111/1759-7714.70145)
Supplement: Supplementary file 1 — Figure S1: Correlations between skeletal muscle index and body weight, body mass index, and psoas muscle index before chemoradiation therapy or durvalumab treatment. BMI, body mass index; PMI, psoas muscle index; SMI, skeletal muscle index. [file TCA-16-e70145-s002.pptx]

## Slide 1
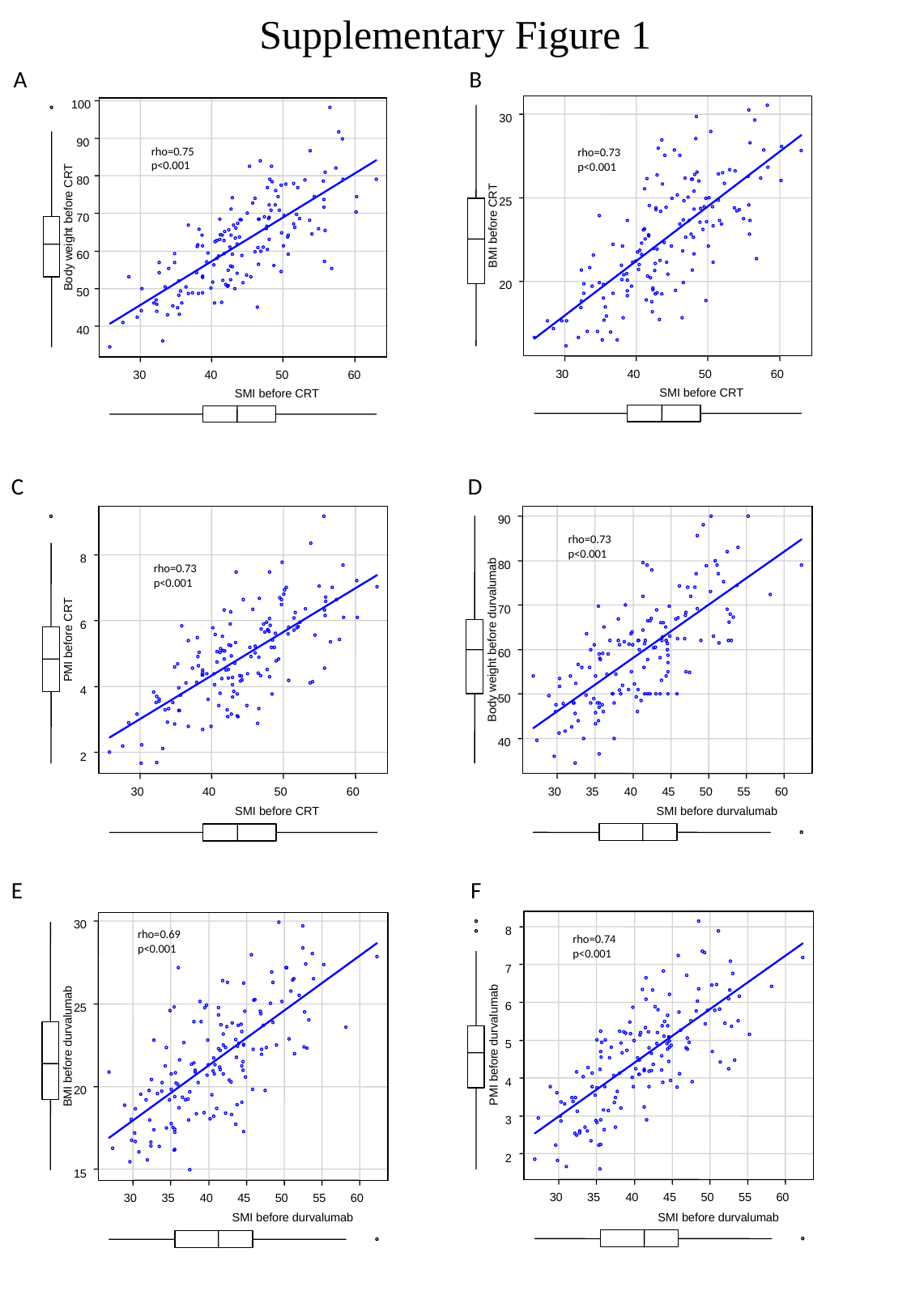

Supplementary Figure 1
A
B
100
90
80
70
Body weight before CRT
60
50
40
30
40
50
60
SMI before CRT
30
25
BMI before CRT
20
30
40
50
60
SMI before CRT
rho=0.75
p<0.001
rho=0.73
p<0.001
C
D
8
6
PMI before CRT
4
2
30
40
50
60
SMI before CRT
90
80
70
Body weight before durvalumab
60
50
40
30
35
40
45
50
55
60
SMI before durvalumab
rho=0.73
p<0.001
rho=0.73
p<0.001
E
F
8
7
6
5
PMI before durvalumab
4
3
2
30
35
40
45
50
55
60
SMI before durvalumab
30
25
BMI before durvalumab
20
15
30
35
40
45
50
55
60
SMI before durvalumab
rho=0.69
p<0.001
rho=0.74
p<0.001
