# Supplementary material for: Effects of Sarcopenia on the Outcomes and Safety of Chemoradiotherapy Followed by Durvalumab for the Treatment of Patients With Locally Advanced Non‐Small Cell Lung Cancer
Source: Thorac Cancer. 2025 Aug 15;16(16):e70145. doi: 10.1111/1759-7714.70145 (PMC12355038; doi:10.1111/1759-7714.70145)
Supplement: Supplementary file 2 — Figure S2: Survival outcomes for patients with locally‐advanced non‐small cell lung cancer treated with chemoradiation therapy followed by durvalumab. Kaplan–Meier estimates of progression‐free survival (PFS) (A) and overall survival (OS) (B) in the entire cohort. PFS (C) and OS (D) subgroup analyses. ALK, anaplastic lymphoma kinase; BMI, body mass index; BW, body weight loss of 5% or higher (ΔBW ≤ −5%) during CRT; CCI, Charlson Comorbidity Index; CI, confidential intervals; EGFR, epidermal growth factor receptor; HR, hazard ratio; NR, not reached; PD‐L1, programmed death‐ligand 1; PMI, psoas muscle index, calculated as psoas muscle area divided by height squared; SMI, skeletal muscle index, calculated as skeletal muscle area divided by height squared; Sq, squamous cell carcinoma. Cachexia was assessed using Evans' definition. [file TCA-16-e70145-s003.pptx]

## Slide 1
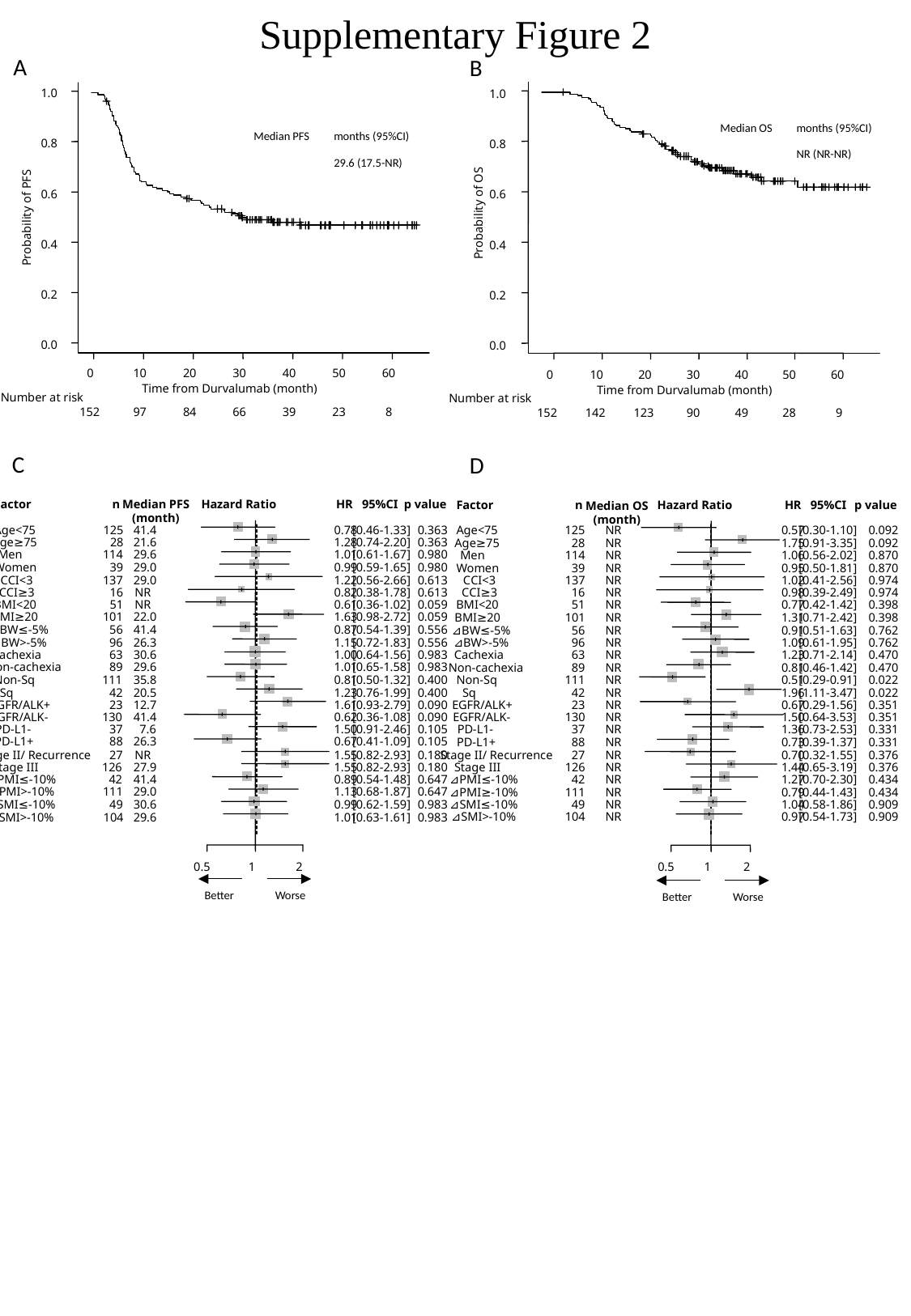

Supplementary Figure 2
A
B
1.0
0.8
0.6
Probability of PFS
0.4
0.2
0.0
0
10
20
30
40
50
60
Time from Durvalumab (month)
Number at risk
152
97
84
66
39
23
8
1.0
0.8
0.6
Probability of OS
0.4
0.2
0.0
0
10
20
30
40
50
60
Time from Durvalumab (month)
Number at risk
152
142
123
90
49
28
9
| Median OS | months (95%CI) |
| --- | --- |
| | NR (NR-NR) |
| Median PFS | months (95%CI) |
| --- | --- |
| | 29.6 (17.5-NR) |
C
D
Factor
n
Hazard Ratio
HR
Median OS
(month)
Age<75
125
NR
0.57
Age≥75
28
NR
1.75
Men
114
NR
1.06
Women
39
NR
CCI<3
137
NR
CCI≥3
16
NR
BMI<20
51
NR
BMI≥20
101
NR
⊿BW≤-5%
56
NR
⊿BW>-5%
96
NR
Cachexia
63
NR
Non-cachexia
89
NR
Non-Sq
111
NR
Sq
42
NR
EGFR/ALK+
23
NR
EGFR/ALK-
130
NR
PD-L1-
37
NR
PD-L1+
88
NR
Stage II/ Recurrence
27
NR
Stage III
126
NR
⊿PMI≤-10%
42
NR
⊿PMI≥-10%
111
NR
⊿SMI≤-10%
49
NR
⊿SMI>-10%
104
NR
0.5
1
2
95%CI
p value
[0.30-1.10]
0.092
[0.91-3.35]
0.092
[0.56-2.02]
0.870
0.95
[0.50-1.81]
0.870
1.02
[0.41-2.56]
0.974
0.98
[0.39-2.49]
0.974
0.77
[0.42-1.42]
0.398
1.31
[0.71-2.42]
0.398
0.91
[0.51-1.63]
0.762
1.09
[0.61-1.95]
0.762
1.23
[0.71-2.14]
0.470
0.81
[0.46-1.42]
0.470
0.51
[0.29-0.91]
0.022
1.96
[1.11-3.47]
0.022
0.67
[0.29-1.56]
0.351
1.50
[0.64-3.53]
0.351
1.36
[0.73-2.53]
0.331
0.73
[0.39-1.37]
0.331
0.70
[0.32-1.55]
0.376
1.44
[0.65-3.19]
0.376
1.27
[0.70-2.30]
0.434
0.79
[0.44-1.43]
0.434
1.04
[0.58-1.86]
0.909
0.97
[0.54-1.73]
0.909
Median PFS
(month)
Factor
n
Hazard Ratio
HR
Age<75
125
41.4
0.78
Age≥75
28
21.6
1.28
Men
114
29.6
1.01
Women
39
29.0
CCI<3
137
29.0
CCI≥3
16
NR
BMI<20
51
NR
BMI≥20
101
22.0
⊿BW≤-5%
56
41.4
⊿BW>-5%
96
26.3
Cachexia
63
30.6
Non-cachexia
89
29.6
Non-Sq
111
35.8
Sq
42
20.5
EGFR/ALK+
23
12.7
EGFR/ALK-
130
41.4
PD-L1-
37
 7.6
PD-L1+
88
26.3
Stage II/ Recurrence
27
NR
Stage III
126
27.9
⊿PMI≤-10%
42
41.4
⊿PMI>-10%
111
29.0
⊿SMI≤-10%
49
30.6
⊿SMI>-10%
104
29.6
0.5
1
2
95%CI
p value
[0.46-1.33]
0.363
[0.74-2.20]
0.363
[0.61-1.67]
0.980
0.99
[0.59-1.65]
0.980
1.22
[0.56-2.66]
0.613
0.82
[0.38-1.78]
0.613
0.61
[0.36-1.02]
0.059
1.63
[0.98-2.72]
0.059
0.87
[0.54-1.39]
0.556
1.15
[0.72-1.83]
0.556
1.00
[0.64-1.56]
0.983
1.01
[0.65-1.58]
0.983
0.81
[0.50-1.32]
0.400
1.23
[0.76-1.99]
0.400
1.61
[0.93-2.79]
0.090
0.62
[0.36-1.08]
0.090
1.50
[0.91-2.46]
0.105
0.67
[0.41-1.09]
0.105
1.55
[0.82-2.93]
0.180
1.55
[0.82-2.93]
0.180
0.89
[0.54-1.48]
0.647
1.13
[0.68-1.87]
0.647
0.99
[0.62-1.59]
0.983
1.01
[0.63-1.61]
0.983
Better
Worse
Better
Worse
