# Supplementary material for: Effects of Sarcopenia on the Outcomes and Safety of Chemoradiotherapy Followed by Durvalumab for the Treatment of Patients With Locally Advanced Non‐Small Cell Lung Cancer
Source: Thorac Cancer. 2025 Aug 15;16(16):e70145. doi: 10.1111/1759-7714.70145 (PMC12355038; doi:10.1111/1759-7714.70145)
Supplement: Supplementary file 3 — Table S1: Changes in body composition during chemoradiotherapy. Table S2: Best responses to durvalumab treatment. Table S3: Comparison of pre‐chemoradiotherapy characteristics between patients who showed decreased SMI during chemoradiotherapy and patients who did not. Table S4: Summary of immune‐related adverse events in patients who showed skeletal muscle loss during chemoradiation therapy and patients who did not. Table S5: Summary of immune‐related adverse events categorized according to subcutaneous adipose tissue index, visceral adipose tissue index, and sex. [file TCA-16-e70145-s001.docx]

**Supplementary Table 1. Changes in body composition during chemoradiotherapy**

| n (%) | Total  (n=153) | Men  (n=114) | Women  (n=39) | p value |
| --- | --- | --- | --- | --- |
| ΔBW≤-5% | 56 (36.6) | 44 (38.9) | 12 (30.8) | 0.443 |
| BMI<20 (kg/m^2^) | 51 (33.3) | 30 (26.5) | 21 (53.8) | 0.003 |
| ΔSMI≤-10% | 49 (32.0) | 35 (30.7) | 14 (35.9) | 0.556 |
| ΔPMI≤-10% | 42 (27.5) | 32 (28.1) | 10 (25.6) | 0.838 |

BMI, body mass index before durvalumab; ΔBW≤-5%, body weight loss of 5% or higher during chemoradiotherapy; ΔPMI≤-10%, a 10% or higher reduction in psoas muscle index during chemoradiotherapy; ΔSMI≤-10%, a 10% or higher reduction in skeletal muscle index during chemoradiotherapy

**Supplementary Table 2. Best responses to durvalumab treatment**

| n (%) | Total  n=153 | ΔSMI≤-10%  n=49 | ΔSMI>-10%  n=104 | p value |
| --- | --- | --- | --- | --- |
| Complete response | 11 (7.2) | 3 (6.1) | 8 (7.7) | 0.756 |
| Partial response | 103 (67.3) | 33 (67.3) | 70 (67.3) |  |
| Stable disease | 18 (11.8) | 7 (14.3) | 11 (10.6) |  |
| Progressive disease | 18 (11.8) | 4 (8.2) | 14 (13.5) |  |
| Not evaluated | 3 (1.9) | 2 (4.1) | 1 (0.9) |  |
| Overall response rate | 114 (76.0) | 36 (76.6) | 78 (75.7) | 1.000 |

ΔSMI≤-10%, a 10% or higher reduction in skeletal muscle index during chemoradiotherapy.

**Supplementary Table 3. Comparison of pre-chemoradiotherapy characteristics between patients who showed decreased SMI during chemoradiotherapy and patients who did not**

| n (%) | ΔSMI≤-10%  n=49 | ΔSMI>-10%  n=104 | p value |
| --- | --- | --- | --- |
| Age (years), median (IQR) | 67.0 [56.0, 72.0] | 64.0 [56.0, 72.0] | 0.315 |
| ≥75 | 10 (20.4) | 18 (17.3) | 0.659 |
| Sex |  |  | 0.556 |
| Men | 14 (28.6) | 25 (24.0) |  |
| Women | 35 (71.4) | 79 (76.0) |  |
| Charlson comorbidity index |  |  | 0.586 |
| 0-2 | 43 (87.8) | 94 (90.4) |  |
| ≥3 | 6 (12.2) | 10 (9.6) |  |
| Cachexia | 10 (20.4) | 20 (19.2) | 1.000 |
| Histology |  |  | 0.908 |
| Adenocarcinoma | 28 (57.1) | 63 (60.6) |  |
| Squamous | 14 (28.6) | 28 (26.9) |  |
| NSCLC, NOS | 7 (14.3) | 13 (12.5) |  |
| Oncogenic driver genes |  |  |  |
| *EGFR* |  |  | 0.579 |
| Negative | 31 (63.3) | 65 (62.5) |  |
| 19del/L858R | 4 (8.2) | 9 (8.6) |  |
| Uncommon | 3 (6.1) | 1 (1.0) |  |
| NE | 11 (22.4) | 29 (27.9) |  |
| *ALK* |  |  | 0.661 |
| Negative | 35 (71.4) | 5 (4.8) |  |
| Positive | 1 (2.0) | 68 (65.4) |  |
| NE | 13 (26.6) | 31 (29.8) |  |
| PD-L1 TPS (%) |  |  | 1.000 |
| <1 | 12 (24.5) | 25 (24.0) |  |
| 1- | 28 (57.1) | 60 (57.7) |  |
| NE | 9 (18.4) | 19 (18.3) |  |
| Stage |  |  | 0.363 |
| IIA/IIB/recurrence | 0 (0.0)/2 (4.1)/9 (18.4) | 2 (1.9)/2 (1.9)/12 (11.5) |  |
| IIIA/IIIB/IIIC | 15 (30.6)/17 (34.7)/6 (12.2) | 39 (37.5)/38 (36.5)/11 (10.6) |  |
| ECOG-PS |  |  | 0.320 |
| 0-1 | 48 (98.0) | 104 (100) |  |
| 2 | 1 (2.0) | 0 (0.0) |  |
| Chemotherapy regimen |  |  | 0.941 |
| CDDP doublet | 39 (79.6) | 80 (76.9) |  |
| CBDCA doublet | 8 (16.3) | 20 (19.2) |  |
| CBDCA | 2 (4.1) | 4 (3.8) |  |
| Radiation dose (Gy), median (IQR) | 60 [60, 60] | 60 [60, 60] | 0.656 |
| Interval between CRT and durvalumab (day), median (IQR) | 19 [15, 24] | 17 [14, 23] | 0.324 |

ALK, anaplastic lymphoma kinase; CBDCA, carboplatin; CDDP, cisplatin; CRT, chemoradiation therapy; ECOG-PS, Eastern Cooperative Oncology Group-performance status; EGFR, epidermal growth factor receptor; Gy, gray; IQR, interquartile range; L858R, exon 21 on L858R mutation; NOS, not otherwise specified; NE, not evaluated; NSCLC, non-small cell lung cancer; PD-L1 TPS, programmed cell death-ligand tumor proportion score; SMI, skeletal muscle index; del, exon 19 deletion; ΔSMI≤-10%, 10% or more loss of SMI during chemoradiotherapy.

**Supplementary Table 4 Summary of immune-related adverse events in patients who showed skeletal muscle loss during chemoradiation therapy and patients who did not**

| n (%) | ΔSMI≤-10%  n=49 | ΔSMI>-10%  n=104 | p value |
| --- | --- | --- | --- |
| Any irAEs | 22 (44.9) | 46 (44.2) | 1.000 |
| Grade 3-5 | 1 (2.0) | 6 (5.8) | 0.431 |
| Require corticosteroid | 12 (24.5) | 35 (33.7) | 0.268 |
| Pneumonitis |  |  |  |
| Any grades | 15 (30.6) | 34 (32.7) | 0.854 |
| Grade 3-5 | 1 (6.7) | 4 (11.8) | 1.000 |
| Hypothyroidism | 3 (6.1) | 4 (3.8) | 0.681 |
| Skin reactions | 1 (2.0) | 4 (3.8) | 1.000 |
| Myositis | 2 (4.1) | 0 (0.0) | 0.101 |
| Arthritis | 1 (2.0) | 0 (0.0) | 0.320 |
| Renal insufficiency | 1 (2.0) | 0 (0.0) | 0.320 |
| Thrombocytopenia | 0 (0.0) | 1 (1.0) | 1.000 |
| Adrenal insufficiency | 0 (0.0) | 1 (1.0) | 1.000 |

**Supplementary Table 5. Summary of immune-related adverse events categorized according to subcutaneous adipose tissue index, visceral adipose tissue index, and sex**

| n (%) | ΔSAI≥10%  n=45 | ΔSAI<10%  n=108 | p value |
| --- | --- | --- | --- |
| Any irAEs | 15 (33.3) | 53 (49.1) | 0.078 |
| Required corticosteroids | 14 (31.1) | 33 (30.6) | 1.000 |
| n (%) | ΔVAI≥10%  n=46 | ΔVAI<10%  n=107 | p value |
| Any irAEs | 19 (41.3) | 49 (45.8) | 0.723 |
| Required corticosteroids | 17 (37.0) | 30 (28.0) | 0.339 |
| n (%) | Women  n=39 | Men  n=114 | p value |
| Any irAEs | 16 (41.0) | 52 (45.6) | 0.710 |
| Required corticosteroids | 13 (33.3) | 34 (29.8) | 0.691 |

irAEs, immune-related adverse events; SAI, subcutaneous adipose tissue index; VAI, visceral adipose tissue index
